# An Audit of the Completion of Physical Health Investigations for Patients Following Admission to the General Adult Inpatient Wards in Mersey Care NHS Foundation Trust

**DOI:** 10.1192/bjo.2026.11697

**Published:** 2026-06-30

**Authors:** Natasha Hall, Beth Jones, Seerat Shirazi, Rachel Rosewicz, Declan Hyland

**Affiliations:** Mersey Care NHS Foundation Trust, Liverpool, United Kingdom

## Abstract

**Aims:**

Individuals with severe and enduring mental illness (SMI) are at greater risk of poor physical health than the general population and have a higher premature mortality rate.Average life expectancy of individuals with SMI is ~15 - 20 years shorter than the general population, often due to physical disease.A significant contributor is underperformance of physical examination, assessment and intervention.NICE Clinical Guidelines and Mersey Care NHS Foundation Trust guidelines state that, following admission to a mental health inpatient unit, a patient should have a physical examination and assessment completed within 24 hours.

This audit assessed if the following were completed within 24 hours of admission to the Trust’s general adult inpatient wards: FBC; U and Es; LFTs; TFTs; bone profile; Vitamin B12 level, serum folate level; serum prolactin level; Hbs-656c level; random serum total cholesterol level; ECG; urine dipstick, urine pregnancy test in females of childbearing potential; and QRISK score in relevant patients.

**Methods:**

A retrospective audit was conducted, with the sample comprising all patients on 12 of the Trust’s general adult inpatient wards on a specific date in December 2025.An audit tool was designed with standards based on NICE Clinical Guidelines and the Trust’s physical health policy and completed for each patient using the patient’s electronic patient record.

**Results:**

129 of the 200 patients were male, 71 female (3 patients were born biologically male but transitioned to female).Age of patients ranged from 18 to 73 years.Within 24 hours of admission the following were completed: FBC - 46%; U and Es - 45%;LFTs - 44%; TFTs - 40%; bone profile - 44%; Vitamin B12 level - 38%; serum folate level - 37%; serum prolactin level - 33%; Hbs-656c level - 39%; and random serum total cholesterol level - 43%.47% of patients had an ECG done.28% had a urine dipstick; 23% of female patients of childbearing potential had a urine pregnancy test.2% of applicable patients had a QRISK score.

**Conclusion:**

Admission to the ward presents an opportunity to “screen and intervene” in a patient population with poorer physical health than the general population and who are less likely to engage in physical health monitoring in the community.An ECG is required if psychotropic medication is initiated or changed. There is a need for greater awareness from both medical and nursing staff on the importance of relevant physical health investigations being completed following admission to the ward.

